# Cotreatment of Congenital Measles with Vitamin A and Intravenous Immunoglobulin

**DOI:** 10.1155/2014/234545

**Published:** 2014-12-07

**Authors:** Yasemin Ozsurekci, Ates Kara, Cihangul Bayhan, Eda Karadag Oncel, Sahin Takci, Sultan Yolbakan, Ayse Korkmaz, Gulay Korukluoglu

**Affiliations:** ^1^Hacettepe University Medical Faculty, Pediatric Infectious Diseases Unit, Sıhhıye, 06100 Ankara, Turkey; ^2^Department of Neonatology, Hacettepe University Faculty of Medicine, Ankara, Turkey; ^3^Turkey Public Health Agency, Virology Reference and Research Laboratory, Ankara, Turkey

## Abstract

Although the measles vaccine has been part of routine national childhood vaccination programs throughout Europe, measles remains a public health concern. High numbers of cases and outbreaks have occurred throughout the European continent since 2011, and an increasing number of cases have been reported in Turkey since 2012. During a recent measles outbreak in Turkey, 2 pregnant women contracted measles prior to delivering preterm infants at Hacettepe University Hospital. Measles virus genomic RNA and IgM antibodies against measles were detected in the cord blood of infants and mothers in both cases. The infants were treated with intravenous immunoglobulin (IVIG) and vitamin A. Transient thrombocytopenia was present in 1 infant and treated with an additional dose of IVIG and vitamin A. The infants were discharged, without complications, within 10 days of birth. The successful treatment of these cases suggests that infants who have been exposed to, or infected with, measles may benefit from cotreatment of vitamin A and IVIG.

## 1. Introduction

Prior to the introduction of measles vaccination programs, a majority of individuals contracted the measles virus early in childhood and developed lifelong immunity; thus, measles infection during pregnancy was an unusual event. The distribution of infected individuals has shifted to older age groups in the vaccine era, with proportionately more adolescent and young adults affected owing to a lack of complete vaccination coverage as well as primary and secondary vaccine failure. The potential risk for measles infection in women of childbearing age has therefore increased [1]. Maternal as well as fetal morbidity increases when measles occurs during pregnancy because it can lead to high rates of fetal loss and prematurity. In addition, pregnant woman has a high risk of severe respiratory distress that might cause death [2]. However, no specific antiviral therapy is available to naïve pregnant women or neonates exposed to the measles virus. Anselem et al. [3] propose that these individuals receive immunoglobulin prophylaxis within 6 days of contact in order to reduce the risk of infection and severe morbidity. In addition, several studies have reported that infected children who receive vitamin A supplements have better outcomes than those who do not [4, 5]. Although there are some reports regarding the effectiveness of intravenous immunoglobulin (IVIG) treatment to prevent or modify disease, there is no data on the combined treatment of congenital measles with vitamin A and IVIG.

At the end of 2011, measles resurged throughout Turkey and further increased during 2012 through May 2013. At the time of this publication, 4,172 cases have been reported, constituting an epidemic. In this case report, we describe 2 preterm infants who contracted measles during this epidemic and their successful treatment with combined IVIG and vitamin A.

## 2. Case Report

**Figure 1:**
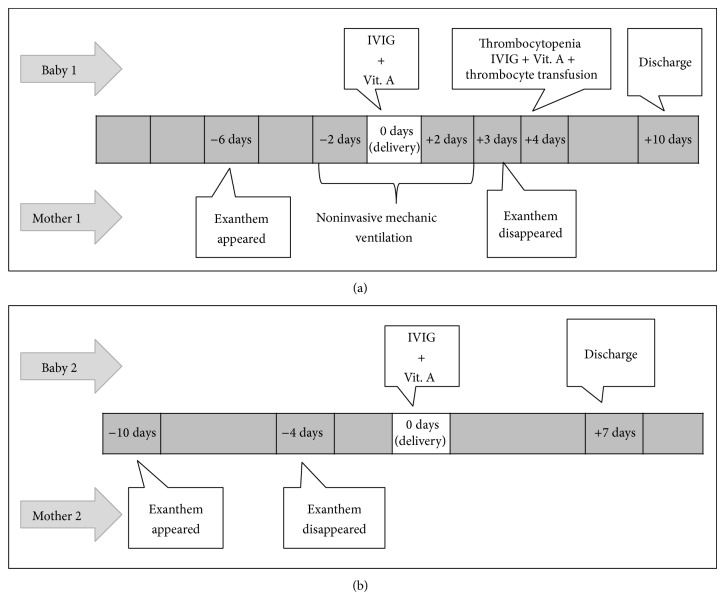
Clinical follow-up of infants and mothers described in Case 1 (a) and Case 2 (b).

Two mothers who had contracted measles during an outbreak gave birth to preterm infants at Hacettepe University Hospital in Ankara, Turkey, in February 2013. We describe both cases and their course of treatment below and in Figure 1.

### 2.1. Case 1

**Figure 2:**
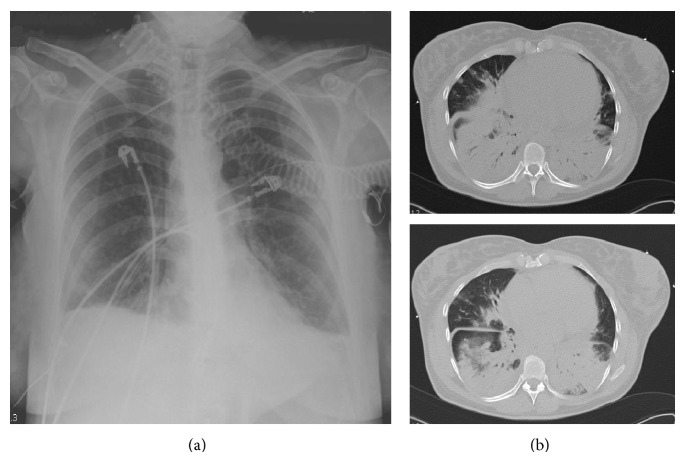
Posteroanterior lung radiograph of the mother described in Case 1 on the day of delivery (a) and computed tomography of the thorax on the day after delivery (b).

A preterm female infant (weight 2780 g) was delivered by caesarean section at 34^1/7^ weeks, because of fetal distress, from a 38-year-old mother with measles pneumonitis. She was born on the sixth day after her mother's exanthema necessitated noninvasive ventilation because of severe pneumonia (Figure 2). The mother's medical history indicated that she had not been vaccinated for measles. The infant showed no clinical signs of the disease at birth.

Cord blood and serum samples were collected from the infant and mother soon after delivery. An enzyme-linked immunosorbent assay (ELISA) indicated the presence of measles-specific IgM and IgG antibodies in the mother. The infant's serum was positive by ELISA for the presence of measles specific IgM but negative for the presence of IgG antibodies. However, RNA extracted from cord blood mononuclear cells for use in a measles virus-specific reverse transcriptase-PCR assay indicated the presence of measles virus (Table 1). On her first day of life, the infant received IVIG (400 mg/kg) and 50,000 IU of vitamin A orally. The initial thrombocyte count shortly after birth was 228,000/*μ*L and had dropped to 44,000/*μ*L after 4 days (Table 1). Thrombocytopenia was further improved after a second dose of IVIG (400 mg/kg) and 50,000 IU of vitamin A orally with the addition of 10 mm^3^/kg thrombocyte transfusion. The newborn was discharged in a good condition on the tenth day.

### 2.2. Case 2

A preterm female infant (weight 2630 g) was delivered by vaginal route from a 27-year-old mother with measles pneumonitis at 36 weeks, on the fourth day after her mother's exanthema had disappeared. The mother's medical history indicated that she had received only 1 dose of the measles vaccine, and the serum obtained 1 month previously was negative by ELISA for antimeasles IgG and IgM. The infant showed no clinical signs of the disease at birth.

Cord blood and serum samples were collected from the infant and mother soon after the delivery and serum specimens were tested for the presence of measles-specific IgM, IgG, and RNA as described above. The infant's serum was positive by ELISA for both measles-specific IgM and IgG antibodies. Measles-virus-specific RNA extracted from cord blood mononuclear cells was positive as well (Figure 1 and Table 1). The infant was treated with IVIG (400 mg/kg) and 50,000 IU of vitamin A on the first day of life and discharged in a good condition on the seventh day.

## 3. Discussion

The widespread availability of the 2 dose measles vaccine program has led to a marked decrease in the incidence of measles and resulted in less natural boosting of antibody levels. However, depending on the schedule of immunization, immunity declines in late childhood and adolescence leading to gaps in the immunity of adults. Further, measles outbreaks may occur in susceptible individuals despite widespread childhood vaccination [6]. Indeed, measles is reemerging in countries that previously had a low incidence of the disease [7] and it is clear that the incidence of measles has increased in Turkey since the beginning of 2012. Measles during pregnancy is associated with serious complications including pneumonitis, hepatitis, premature labor, abortion, and death. Pneumonitis is the most common and serious maternal complication [8]. The mother described in Case 1 suffered from pneumonitis with severe respiratory distress necessitating noninvasive ventilation during labor and the mother described in Case 2 also suffered from pneumonitis shortly before labor. Consequently, both infants were delivered prematurely without complications.

Primary protection against infectious diseases at birth is provided mainly by maternal antibodies and several factors determine the amount of maternal antibodies in young infants. The coverage of universal immunization programs influences the amount of maternal antibodies and a higher level of coverage reduces the probability of natural antibody boosting. Infants of vaccinated women were born with significantly fewer antibodies compared to infants of naturally immune women. Because increased childbearing age is directly related to the prolongation of the time between childhood vaccination and childbirth, maternal antibodies are prone to be low. The rate of decay of maternal antibodies after birth determines the duration of protection in infants [6, 9–11]. Young infants are protected from measles infection by maternal measles-specific IgG antibodies that are actively transported across the placental barrier beginning at 28 weeks of gestation and their levels increase until the time of birth. The level of these antibodies at birth depends on the level of antibodies in the mother and the extent of placental transfer. Thus, gestational age defines placental transfer; and consequently, preterm infants receive significantly fewer antibodies than those delivered at full-term [12]. Since one of the mothers was unvaccinated with measles vaccine and relatively had old childbearing age (Case 1) and the second mother had received only one dose of the vaccine (Case 2), there is a low probability that the mothers, in this study, have sufficient antibody levels to provide protection against contracting measles. Therefore, the negative IgG ELISA finding from the first infant may be attributable to both low gestational age and an inadequate interval of transplacental transfer of maternal measles-specific antibody (which could only have occurred from the onset of maternal exanthema until the time of the infant's delivery). Positive IgM/G ELISA of the second infant might be explained by the enough transmission time of protective maternal antibodies from mother to infant. In both cases, delivery was postponed as long as possible to allow maternal antibody transfer.

The effect of measles during pregnancy on the fetus has been the focus of several studies. Limited data is available regarding the spectrum of neonatal illness, but it can range from mild to rapidly fatal forms. Early studies have documented adverse fetal outcomes including increased mortality during the first two years of life, and it appears that the mortality rate of congenital measles is higher in preterm than in full-term infants [1, 8]. Interestingly, hematological manifestations of congenital measles have not been previously reported and to the best of our knowledge, this study is the first report of a case with thrombocytopenia due to congenital measles.

The low morbidity of recent cases of congenital measles may reflect the effects of prophylactic immunoglobulin [1]. These two cases of congenital measles may further encourage the prophylactic use of immunoglobulin in all exposed infants, as recommended by Chiba et al. [1]. Although there is limited data regarding the necessity and dose of vitamin A for the treatment of neonatal measles, the cases described in this report were treated with 50,000 IU of vitamin A in addition to IVIG. Vitamin A deficiency has been associated with severe cases of measles in children in developing countries. Acute measles precipitates vitamin A deficiency by depleting vitamin A stores through an increased systemic utilization. Treatment with vitamin A reduces morbidity and mortality in measles, and all children with severe measles should be given vitamin A supplements, whether or not they are thought to have a nutritional deficiency [4, 5].

In conclusion, the two cases of congenital measles reported here highlight the need to improve immunization strategies for adolescents and young adults, which could decrease the incidence of maternal measles. Additionally, the survival of these preterm infants suggests that cotreatment with vitamin A and IVIG is a successful strategy for vulnerable individuals who have low levels of protective measles antibodies.

## Figures and Tables

**Table 1:** Laboratory findings of mothers and neonates.

Case	Age	Sample taken (after date of birth)	Platelet count (/*μ*L)	ELISA		Cord blood PCR
				IgG	IgM	
1	Newborn (preterm, 2780 g, C/S^*^)	Day 1	228.000	(−)	(+)	(+)
		Day 4	44.000			
		Day 7	273.000			

1	Mother 38 years	Day 1	305.000	(+)	(+)	

2	Newborn (preterm, 2630 g, VB^**^)	Day 1	217.000	(+)	(+)	(+)
		Day 7	295.000			

2	Mother 27 years	Day 1	208.000	(+)	(+)	

^*^C/S: caesarean section; ^**^VB: vaginal birth.
